# Health care assistant and registered nurse dyads, working together and apart – a qualitative study

**DOI:** 10.1186/s12912-024-02619-z

**Published:** 2024-12-30

**Authors:** Rachael E. Carroll, Kay De Vries, Claire Goodman, Jayne Brown

**Affiliations:** 1 Injury, Recovery & Inflammation Sciences, University of Nottingham, Nottingham, UK; 2 Leicester School of Nursing and Midwifery, De Montfort University, Leicester, UK; 3 Centre for Research in Public Health and Community Care, University of Hertfordshire, Hatfield, UK; 4 Institute of Health and Allied Professions, Nottingham Trent University, Nottingham, UK

**Keywords:** Nursing, Team, Wards; general, Healthcare assistants, Medical-surgical nursing, Task performance, Dyad, Ethnography, Professionalisation, Person-centred

## Abstract

**Background:**

This study was undertaken to understand the role of the Health Care Assistants and how they negotiate roles and responsibilities with Registered Nurses in adult acute hospitals.

**Methods:**

The qualitative approach of focused ethnography used non-participant observation and interviews with staff from four acute wards. Field notes and interview data, analysed using NVIVO10, moved data from description through explanation, interpretation and identification of themes.

**Results:**

148 h of observations and 108 interviews were conducted in dyads comprising 22 Health Care Assistants and 33 Registered Nurses. Health Care Assistants worked non-dependently from, and inter-dependently with Registered Nurse dyad partners. Dyads relied on demarcation of responsibilities by task, established and reinforced by ward culture. Demarcation enabled Registered Nurses to oversee care but could create false divides between observing and recording patients’ conditions and interpreting findings. Interdependent working only happened when two staff members were needed for care. Involvement in fundamental care by the Registered Nurse was unpredictable and discretionary. There was limited evidence of how dyads supported person-centred approaches.

**Conclusion:**

The physically-boundaried, close working of the Health Care Assistant and Registered Nurse had an isolating, task driven impact on Health Care Assistants’ work. Recognising the dyad did not foster shared goals, learning or review of care.

## Background

Unregistered staff work alongside Registered Nurses (RNs) to provide patient care [1]. Internationally, many titles are used to describe this role including Assistants in Nursing in Australia, Unlicensed Assistive Personnel and Nursing Aide in the USA, and Health Care Assistants (HCAs) in the UK [2]. In August 2023, HCAs were amongst the 341,033 full time equivalent support staff working in the UK’s National Health Service (NHS) compared with 374, 137 RNs and Health Visitors [3].

The roles and responsibilities of HCAs as members of the nursing team have changed alongside the scope of the RN. The original model of delivering care was based on one member of the ward nursing team doing the same task for all patients. Those in subordinate roles, such as HCAs, were ‘bed-makers’ and ‘temperature takers’ [4]. This was replaced by a team nursing model where the whole ward team were divided into smaller groups of nursing staff. Each team member carried out more tasks for fewer patients which, it was argued, resulted in staff knowing each patient better and improved quality of care [5]. This developed into the patient allocation or primary nursing model, with each RN responsible for all the healthcare needs of a small number of patients; a method of organising nursing work that claims to be closer to the tenets of person-centred care but has limited evidence of improved patient outcomes [6, 7]. Gillies’ [5] description of the primary nursing model did not consider the contribution of the HCA even though they are a workforce consistently identified as the providers of fundamental care [8, 9]. This omission is suggestive that nursing undervalues this aspect of care work [8, 10].

Continuing to flex in response to changes to nursing roles, in 2000, the Department of Health [11] recommended that the HCA role was extended in acute care and introduced in other settings to absorb what historically had been defined as nursing work. Studies in critical care [12], intensive care [13], and community nursing [14], suggested this has resulted in blurred occupational boundaries, unclear job descriptions and issues with standardisation of training. RNs described being anxious about delegation and accountability, and some saw changes in HCA practice as a threat to their own role [9, 15]. The impact of these changes was arguably not formally discussed in England as an issue for patient safety until a national inquiry into avoidable deaths in one hospital raised it as an issue [16].

The lack of registered health care staff, the role expansion for RNs and junior doctors, as well as cost containment, has led to the HCAs’, or nursing assistants’, scope of practice increasing [2]. What is and isn’t nursing work is not fixed and reflects how roles and expertise are recognised and supported [17]. There is compelling evidence of the negative effects for patients when staffing levels of RNs are reduced and HCAs compensate for shortages of RNs [18, 19]. The potential dangers of the unsupervised HCA role in care delivery are recognised in the literature on patient safety and risk management [16]. Equally, the systemic exploitation and failure to value the HCA’s emotional labour as a front-line provider of complex and challenging care has been documented [8]. From the PhD thesis of author REC [20], this paper focuses on the working relationships of HCAs with RNs and how these are negotiated and organised in acute care wards.

Research on nursing skill mix have either not included the voice of the HCA, for example where the focus was on manager’s viewpoint [14], or their experiences conflated with those of Registered Nurses [13]. Yet their competence has been seen as a particular strength [9].

### Aim and objectives

The aim of this study was to explore how HCAs enacted their role in an acute adult, in-patient, hospital environment.

The objectives were:

To understand how HCAs connected, interacted, and related to RNs and other ward-based staff.

To understand how their different working interactions shaped their understanding of their role and responsibilities when working with RNs.

## Methods

### Design

This ethnographic study aimed to explore the relationships of HCAs within an acute care setting. The philosophical approach of this ethnography is interpretivist and relativist, focusing on understanding cultural practices and meanings within this specific social context, where reality is viewed as constructed through shared experiences and interactions. The study took account of theories on person centred-care, nursing teams, the role and expertise required to meet patients’ complex needs and the literature on fundamental nursing care [10, 21–23]. Data collection and analysis were informed by these theories but the focus became the HCA-RN interactions in achieving the espoused goals of person-centred care.

The qualitative approach used non-participant observation and interviews with staff from four acute wards. Field notes and interview data, analysed using NVIVO10, moved data from description through explanation, interpretation and identification of themes.

To address the exploratory nature of the study, focused ethnography [24, 25] was used to look at the context of care and what the interactions of the participants in the study revealed about how roles were framed and understood. This research approach favours researcher practitioners as it assumes that the researcher has prior knowledge of the setting to facilitate data collection and (in this case) application to practice [24, 25].

It is recommended that the researcher carry out reconnaissance work [26]. Apart from the logistical element of exploring the environment and identifying possible gatekeepers, this included speaking to possible participants, spending time observing and looking at significant documents. This highlighted a possibility that HCAs may not be adept at verbally recounting what they did or how they knew what to do. This reflected Wolcott’s [26] (p74) observation that “culture is mostly caught rather than taught”. To understand their work, the researcher (REC) observed HCAs working in patient bays and followed this immediately with an interview taking examples from what had been observed to explore their decisions and actions.

### Setting

The study took place in one UK hospital, in four participating wards; two medical and two assessment wards. The medical wards had 30 beds; four bays with six beds, and single occupancy side rooms opposite each bay. One assessment ward had the same lay-out but was double in size. The other assessment ward had sixteen beds and six chairs for short term observation or treatment.

The organisation of nursing care was the same across the four wards. The ward manager and nurse in charge of the shift had an overview of the ward. One HCA and one RN were allocated to work in each bay and two side rooms, having responsibility for up to eight patients, for a twelve-and-a-half-hour day shift. For continuity, the senior nurse on the night shift allocated RNs and HCAs who had worked the previous day shift to the same bay on consecutive days. Nursing staff that had not worked the previous day were slotted into the gaps. HCAs often worked with a different RN on each shift. They couldn’t see other HCAs and RNs when working in their allocated patient bay.

### Participant recruitment and data collection

Purposive recruitment of HCAs was through the four ward managers who, as gatekeepers, shared information about the study and introduced the HCAs who expressed interest to the researcher. Face to face introductory meetings occurred, where aims, reasons for undertaking the study, and rights to withdraw were discussed before consent was taken. It was made clear to staff that whilst gatekeepers gave permission for the researcher to be present on the ward, they did not have to participate. Observations in the bay where the HCA was allocated to work the shift lasted approximately four hours, ending when there was a natural break in the workload. The observer (REC) sat at the entrance to the bay and made field notes, with pencil and small note pad, in real time and did not engage with the activities or participants [27]. There was no preset structure or codes to record observations. To ensure data quality, all observations were typed in full by the researcher within 24 h of data collection [27]. Researchers’ reflections were added to a column next to the transcription at this time.

The combination of observation, followed immediately by an interview, meant it was possible to clarify and discuss the activities observed. An interview guide was not used. This approach to data collection supported understanding of the content of an interaction, why actions were taken, and the participant’s interpretation of these events. At times, early analysis was shared and discussed to check and develop understanding. After each period of observation and interview of the HCA, the RN that was working with them in the bay for the shift was invited to interview. Some of the same events and interactions were discussed.

To gain a broader prospective of how the HCA role was understood, ward managers were interviewed for their organisational insights. An occupational therapist and a student nurse from each ward were also interviewed which provided a sense-check of the ward culture and how the HCAs role was perceived. Interviews were conducted in a room on the ward and lasted between 21 and 59 min. They were audio-recorded and transcribed verbatim.

The data obtained were reviewed for new insights from interviews and the observed ways of working on the ward. Data saturation occurs when the researcher gathers data to the point of diminishing returns, where nothing new is being added [28]. Data collection ceased when there was sufficient data to test and develop how concepts of relational working were understood, negotiated and provided by the participants within the ward context.

### Data analysis

Analysis was an iterative process beginning with writing up field notes from observations and transcribing interviews [29]. Coding was carried out by REC and discussed with other authors. Analytical memos noted connections between observations and interviews or between data sets, different HCAs in the same setting, commonalities and differences between HCAs on different wards, the same HCA at different times. Themes derived from the data were captured in mind maps, and NVivo 10 was used to organise and categorise the data.

Focused analysis moved the text from description towards explanation, interpretation and identification of patterns [29]. Drawing on Hammond’s [30] model of theorising, core questions were tested, for example, around how the ward nursing team functioned as a whole when HCAs and RNs in bays couldn’t see each other.

### Rigour and reflexivity

Credibility and transferability of the data were supported by protracted engagement with observations and interviews, member checking during interviews and comparison of data from different sources [30, 31]. Transferability was demonstrated through sharing the research design, recruitment strategy, and detailed description of the setting.

Reflexivity was met and developed through the lead academic (REC) discussing findings and analysis with authors to appraise values and biases from her personal background and mental health nursing experiences.

Ethical approval for this study was granted by West Scotland Research Ethics Service (ref 14/WS/1130). As ethical approval was granted for staff participation only, observation of patient care behind curtains was not undertaken.

### Findings

In total, the combination of observation immediately followed by interview resulted in 148 h of observations from 41 sessions and 108 interviews. Health Care Assistants (*n* = 22) were observed then interviewed between two and four times over twelve months. Contrary to expectations and ward managers endeavouring to maintain continuity by allocating the same staff to the same bay, not once was the same combination of HCA and RN observed. Therefore, a large group of RNs were interviewed (*n* = 33). One RN declined, stating time limitations. Quotations are labelled with the following details: their source (e.g., HCA), type (e.g., interview or observation), the participant’s unique ID number (e.g., 14), and the interview number if there were multiple interviews (e.g., .2). Field notes not tied to a specific individual are labelled “Field note,” followed by a code indicating the location and time.

A HCA and RN paired for a shift formed a team of two. They were responsible for completing and recording all the nursing care of the eight patients in their allocated bay and side rooms throughout the shift. Observation showed that HCAs carried out clinical observations, for example blood pressure, pulse and temperature, and provided fundamental care such as washing, eating and elimination. Central to the provision of care and doing key tasks on time was how cohesive this relationship was. As demonstrated in this quote, successful nursing care was reliant on a HCA who was “good”.

*Very good shifts are when you have a very good team. If you have a very good Health Care Assistant*,* your shift just goes amazingly (RN int 26).*

Registered Nurses described HCAs as ‘good’ when they were able to work with minimum prompts and required little checking of their work. Although both the HCA and the RN had individual tasks, such as washes and medication respectively, they also needed the support of each other to complete the work. Their work was entwined and differentiating what was and was not HCA work, what required supervision or collaboration, and what could be enacted alone depended on a set of characteristics. These were: how they came together, where prior experience of working together was a pre-cursor for how the shift might feel; division of work, describing the organisation of particular tasks; focus on each other, being physically isolated from other HCA-RN dyads and only having each other for support. The analysis focused on the HCA and their interactions with RNs as a dyad, working together and apart.

### Form of HCA-RN dyad

#### HCA-RN dyad coming together

When HCAs knew which RN they were paired with, this affected their preconceptions and if there was “gelling”. They described two interlinked processes that drew on ideas of personal compatibility and capacity to complete the work. Pre-shift preconceptions were expectations for the quality of the partnership, based on the RNs’ reputation and the individual’s previous experiences of working with the person. It brought to the fore possible adjustments that the HCA might make, for example, anticipating they would need to ask for help outside of the dyad. “Gelling” was a term used by a HCA to describe the development of a positive relationship between the pair. It did not reference particular skills or the absence or presence of expertise. Rather, it was how they took opportunities to learn each other’s personal characteristics. During the observations this was seen as an unplanned and ad hoc process but was important in determining how confident the HCA would be in asking for help.

*It is nice when you get that five minutes to sit down because you get to have a chat with [RN] about other things than what is going on in the bay. You get to know them a little bit (HCA Int 22.2)*.

*…that you can get on professionally*,* it’s important […]. She is more likely to ask for help*,* I’m more likely to ask her for help*,* instead of it being a bit awkward (RN Int 19.2).*

When a dyad had not invested time to develop a positive relationship or someone was known to be difficult to work with, pre-shift preconceptions were more likely to be negative and there was acceptance that there was a difficult shift ahead. The pairings by senior nurses before commencement of the shift were generally accepted without question. It was difficult for HCAs to question their allocation, which could cause ‘problems’. “*So many people have bad mouthed that person… nothing gets done” (HCA Int 11.4).* To avoid conflict, senior nurses used a technique of “*sharing that person out (LAUGH)”* (HCA Int 11.4) so that no-one worked with them too frequently.

#### The division of work

The hierarchy of registered and unregistered nursing staff was reflected in the tasks HCAs were observed to complete. For example, fundamental care such as washing and helping patients with eating was represented by the RNs as straightforward work and part of the daily routine for the HCA. There was little acknowledgement by the RNs of what it could entail: “*So*,* they actually know what needs to be done and they will go off and do it”* (RN Int 4).

Observations however, suggested that this could be complex and challenging work requiring a range of skills especially for patients living with cognitive impairment or where English isn’t their first language. Often HCAs gave over little of their attention when supporting a patient to eat, possibly due to time pressures (Field note M2 29.06). However, this unusual example below illustrated a new HCA’s cultural sensitivity, her commitment to the goal of care (helping someone to eat) rather than the task alone and crucially seeking advice from another HCA who might have additional insights. None of these activities were seen by or involved the RN.

*“Would you like some dinner?”. She gently touches her cheek with her hand*,* the patient turned towards her. The HCA shows her the soup “do you want some soup?”. She feeds her two spoonful’s then the lady puts her hand up and turns away. The HCA picks up the main course. The patient has four spoonsful of this*,* then does the same. The HCA puts down the dinner and leaves the bedside. She returns with another HCA who speaks the patients’ language. After a short talk*,* the second HCA tells the first HCA to mix the meat with the rice. The patient then ate a great deal more. The HCA rinses the patients’ beaker and refills it with water. After giving her a sip*,* she puts it into the patient’s hand and the patient has a long drink (HCA observation 2.3).*

#### Focus on each other

A distinctive quality of the HCA-RN dyad was their physical isolation, detached from the other HCA-RN dyadic teams on the ward. They did not usually engage with patients or staff in adjacent bays. This physical isolation combined with a focus on completing tasks for a specific group of patients increased the inward-looking nature of the dyad working relationship:

*You have got a nurse and a HCA working together for the full day*,* they know what is happening in their bay*,* they have got their group of patients and that’s it.* (Ward manager Int 14)

The HCA and the RN were isolated from peers, and their relationship with each other was one of possession. They referred to each other as *“my”; “my nurse”*, *“my HCA”*:

*They took my nurse off me in the bay and sent her to another ward* (HCA Int 3.3).

*The HCA told me the RN asked ‘why are you going to help them*,* you are my nursing assistant*,* not theirs’. She replied that they needed a few of us to turn someone* (Field Note A2 26.10).

### Function of HCA-RN dyad

To be considered as successful, the HCA needed to complete and record all the nursing tasks within the shift, many of these tasks were done without reference to the RN. There was a negotiated choreography observed within the HCA-RN dyad, enacted through the joining and separating of the partners. Whilst HCAs carried out tasks without the physical help of the RN, they did not function as a separate entity. Non-dependent working described a range of HCA behaviours applied when carrying out their routine tasks. They had discretion in deciding the order of some of their work, for example when they completed their *“writing”* in the patient’s notes and which patients they prioritised for washing. These decisions were based on which formula was most likely to ensure pre-set tasks were completed by a certain time. HCAs were ‘non-dependent’ rather than ‘independent’.

'Non-dependent’ was a preferable term conceptualised to describe the behaviours of the HCA when carrying out the routine scaffolding. ‘Independent’ is often defined as behaviours of a person who is not influenced by others, does not require others’ support and works autonomously. Whilst accepting that HCAs carried out tasks without the physical help of the RN, they did not function as an entirely separate entity. Their behaviours were interwoven with the RNs actions and preferences. Their ability to work non-dependently in this way was based on a shared expectation or understanding within the dyad, and of knowledge of the tasks to be completed.

This task focused work provided a ‘routine scaffolding’; a conceptualised framework which ensured that all care tasks were completed, in order of priority, for organisation of the twelve and half hour shift. Routine scaffolding incorporated three types of tasks; compulsory timed tasks (e.g. clinical observations), mandatory flexible tasks (e.g. washing and dressing), and RN requested tasks (e.g. additional monitoring).

The compulsory timed tasks took priority for the HCAs. HCAs entered the results of clinical observations onto the National Early Warning System (NEWS) programme via an electronic device such as a tablet or phone. Referred to as *“e-obs”*, when the time was reached for the observations to be repeated, a red clock symbol appeared on the *“e-obs”* device as an alert. The ward manager also received the alerts and would approach the HCA when the red clock appeared to ask why the task was not yet completed. This emphasis and its perceived importance, regardless of the patient condition or risk of decline, was captured through HCAs saying *“obs come first”.*

A HCA was observed going to take a patient’s observations but seemed unhappy with the pulse oximeter. In the interview that followed, she stated that it was measuring 89% saturation. She didn’t think that this was right and was observed to fetch another pulse oximeter to try again. Whilst she was doing this, the medical team arrived at the bedside. The HCA continued to carry out the observations in their presence. In the interview, she explained why she persevered when the doctor commented that the results were fine, implying that she should leave:

*It is because we are constantly getting*,* I don’t know what is the right word*,* under a lot of pressure with obs being on time. His obs were already overdue*,* I think by 5–7 min or something but if I had left it until after the doctors had finished it could have been like 20 min*,* half an hour later and we get in trouble because we have not been on it with the obs. We have been told that obs come before everything* (HCA Int 6.3).

Mandatory flexible tasks were those that needed to be completed but did not have a time to be done by. These included supporting patients with washing and eating and recording these actions in patient’s notes. They were fitted in between the compulsory timed tasks and other known events that could be unpredictable, such as the time the breakfast trolley reached the bay.

*I thought I will get my [laundry] trolley loaded up and get everything prepped ready [for washing the patient] because I knew that there was going to be a problem with giving out breakfasts* (HCA Int 5.3).

These two types of tasks (compulsory timed and mandatory flexible) shaped the routine scaffolding that ensured tasks were less likely to be missed or delayed. Progress throughout the shift was marked by these activities. It was not a concrete procedure that could be enacted without thought or adaptation; each shift was unique in terms of the amount of patient care and the frequency and times of tasks. But these tasks were observed to be completed by all HCAs, in a similar format, on every shift. They were embedded behaviours.

RN requested tasks, such as being asked to undertake an electrocardiogram (ECG) for example were observed to be infrequent and triggered by patient need. They did not alter the requirement to complete compulsory timed tasks and the mandatory flexible tasks:

*If anything needs doing my nurse will tell me what I need to do in the bay that I look after but you kind of go off and do your own thing because you know what you have got to do* (HCA Int 8.3).

This scaffolding work meant the RN was released to carry out tasks that were seen as exclusive to their role. However, the rigid demarcation and focus on the task rather than its implications for patient care meant that decisions about the significance of results could be missed because of lack of RN involvement.

The extent to which the HCA was able to contribute to the overall success of the HCA-RN dyad was dependent on the RN trusting them enough to complete the routine scaffolding. Although HCAs expressed feelings of responsibility, the RN held overall accountability regardless of whether they or the HCA carried out the task. RNs determined which HCAs to trust over time, based on individual HCA ways of working, and identifying their strengths and weaknesses. This assumed that continuity of association was important for HCA-RN working relationships even though this was not guaranteed. This quote demonstrates the additional oversight work of the RN at the beginning of the dyad working relationship:

*Well at first*,* I was just literally checking paperwork all the time like*,* has that person been turned*,* is that done? And eventually you work out who is better at some jobs than others.* (RN Int 15).

When trust was present, partnerships became more intuitive in nature:

*I know that I don’t need to tell her that you need to fill in the score sheet*,* you have forgot to do this and forgot to do that*,* she will do it without me telling her*,* she knows* (RN Int 14).

Non-dependent working was established against a backdrop of predictable actions and trust in HCA responses to task related priorities. In contrast, inter-dependent working occurred when a HCA couldn’t complete a task alone. When this was necessary, the hierarchy was observed to remain; the RN led the interaction with the patient, directed the HCA and instructed follow-up tasks. These ranged from moving patients in the bed to facilitate washing, to involving an RN to assess skin integrity or assist in a procedure.

*The RN irrigated and dressed the pressure area wound whilst the HCA held the patient securely on her side and gave her reassurance* (HCA Observation 4).

Although there were patterns of working that were recognisable as HCA or RN work, communication within the dyad was important. For example, the HCA informed the RN about “obs” for patients that “scored”, meaning their results were outside of set parameters. The RN would then advise the HCA on next actions. This is how they joined and separated over the shift:

*After she gave out the medication*,* she has been communicating with me*,* asking me who’s scoring*,* asking what’s going on*,* asking who needs a hand* (HCA Int 1.3).

At regular intervals, the HCA and the RN would spend a small amount of time together to plan, or review and re-plan actions. These regular “mini-meetings” (RN Int 7.1) and knowing where each other were, meant the HCA could anticipate when they would need an RN for inter-dependent tasks. When this did not happen, it could lead to the HCA being constrained in their work whilst waiting on the RN’s availability. In this example, the HCA was waiting for support in moving a patient from the commode to the bed:

*HCA calls RN by name for a second time*

*HCA - Are you ready?*

*RN – I am coming*

*Daughter – you said two minutes*,* not that we were counting.*

(HCA observation 3.1)

Some HCAs perceived that RNs did not acknowledge the inter-dependence between them, there was an absence of reciprocity and recognition of the HCA’s reliance on the RN to achieve patient care.

*I think it’s all about hierarchy really*,* sadly. Because they are up here*,* they can do everything we can’t do. And they will be like*,* oh*,* we are busy […]. I think there are a lot of nurses that don’t want to help* (HCA Int 11.4).

The RNs could make choices and control elements of their workload. Some RNs actively chose not to provide fundamental care of their patients. HCAs perceived this as meaning that providing fundamental care was not valued. Some were suspicious that some RNs extended the time spent on RN-exclusive tasks as an avoidance strategy, *“two and a half hours for a medication round to do seven patients”*:

*They will hold onto their drugs trolley*,* they will wander off and disappear for God knows how long and they will make no end of different excuses to why they can’t come and help you wash people* (HCA Int 2.4).

HCAs were not in a position to comment on RN decision making. Nor did they have the power to make RNs attend to inter-dependent working tasks if they chose not to. As a dyad, they were observed to be ‘locked’ into a very close working relationship but one that was unequal. This was mitigated by greater length of time working together and greater mutual trust.

How HCAs articulated their role parameters and which tasks they considered to be shared varied. Some HCAs saw their role as clearly demarcated, defined as completion of as much of the work as possible that did not require professional registration. By relieving RNs of tasks, some HCAs believed they created space for the RN to concentrate on higher level tasks. Other HCAs felt this was an elastic concept unrelated to registration, and reflecting how physically demanding the work was. They felt taken advantage of, it was telling that providing patient care was described as “rubbishy”:

*I think some nurses think because you are the HCA you do all the rubbishy jobs and they don’t do that*,* that’s not in their job description* (HCA Int 4.3).

Some HCAs described the strategies they used to ensure the RN also contributed to the direct personal care of patients and limiting the opportunities for being taken advantage of:

*I don’t do no washes until they have done the meds. If you start them*,* they will think “she will carry on*,* so if I just take my time*,* I won’t have to help her” (HCA Int 7.4).*

Success, from the HCA perspective of the HCA-RN dyadic team, occurred when RNs did half of the fundamental care tasks:

*Today went really well because [RN name] fed one patient*,* I fed another*,* […] so that was an equal balance* (HCA Int 1.3).

This quote suggests that one HCA viewed true sharing of this work with the RN as important. A shift went well if the tasks were shared between the RN and the HCA.

## Discussion

Focusing on how RNs and HCAs are allocated to work together in one hospital has provided an in-depth description of a HCA-RN dyad. It illuminates how the roles of registered and unregistered nursing staff are negotiated and enacted, with little or no alteration for individual patient need or diagnosis. We suggest that there is relatively little empirical evidence to support a theory of HCA-RN working. This paper brings new insights to inform further work.

There was an observed and learnt choreography where HCAs connected, interacted, and related to the RN they were paired with for a shift, joining and separating for different aspects of the work. HCAs recognised their reliance, responsiveness and reactions to ‘their’ RN. Each of the partners had a closeness to the other that was not replicated with other people outside of the pairing; the definition of a dyad [32]. In an American study, Kalisch [33] referred to ‘dyads’ (p 20) when identifying seven barriers to nursing team working. Barriers included: lack of role clarity; inability to deal with conflict; and the Unlicenced Assistive Personnel (UAP) not being included in decision making. However, as Kalisch [33] described a higher number of RNs than UAPs on a shift, it suggests they were not dyadic pairs that worked in close proximity, and to the exclusion of all others, as the HCA-RN dyads in this study were. Indeed, it is possible that many of the seven barriers to RN-UAP teamwork identified by Kalisch [33] would be addressed if learning from HCA-RN dyad was successfully implemented.

Kenny and Cook’s [34] dyadic data analysis statistical measures have been used to explore areas in healthcare [35, 36] and sport [37] that go beyond descriptions of delegation and substitution. In the HCA-RN dyad, the members of the partnership change each time they perform. Each person in the dyad has a unique role, which in turn, relies upon the actions of the other. The predictable, trusted behaviour and bay-based presence of the HCA provided a basis for nursing care. Their performance brought stability and predictability to the dyad’s work. It relied on a scaffold of routine and task-based working that allowed minimal deviation from pre-set schedules of observations and ‘care’. Person-centred care, or responses to individual needs aligned with a patient’s condition, were compromised by this approach.

Health Care Assistants through their intimate contact with patients, have the potential to learn patients’ preferences and needs; essential for person-centred care [21, 22]. It is well documented that RNs valued HCA’s focus on fundamental care which developed better connections with patients; something RNs were less able to achieve [8, 9]. However, HCAs in this study did not feel valued and often their interactions were task related instructions. Daykin and Clarke [38] (p356) used the term *“announcements”* to define this form of communication and stated that *“there was no real evidence of patients being involved in decisions about their care”*. Withdrawing from patient contact was a coping strategy used by RNs when fundamental care was not recorded, thereby suggesting it was not valued [21]. HCAs may also be using this strategy; modifying behaviours in order to withdraw from patient interactions [23]. It is possible that the culture of care did not encourage them to engage in a different way with patients. In this situation, the RNs were reinforcing a depersonalising approach.

This study suggests that RNs are more comfortable with HCAs involvement in care delivery than was seen in the earlier literature [12, 15]. The work of HCAs has ceased to trigger any professional dissonance. It may also be that RNs no longer valued fundamental care or see it as their role [21]. Kessler et al. [17] proposed that when RNs view advanced nursing tasks as the centre of nursing, it leads to task orientation and separates them from the patient. This is in direct contrast to a culture where all nursing tasks are considered to be core. It was evident in this study that this task orientated ‘specialist-discard’ [17] approach was in place. The cultural rationale on which RNs based their work was who was “qualified” to complete them. Whilst the findings did identify collegiate working and exchange of information, it was primarily to fulfil a shared understanding of a hierarchy of tasks to be completed around the patient. The experiential knowledge of both HCA and RN and ‘choreography’ of teamwork that could lead to personalised care required a sustained period of working together, something that was not built into how the dyads were organised.

The four adult in-patient wards were self-managed within one hospital Trust. They were required to demonstrate their effectiveness by meeting performance targets using audits [39]. This contrasts with fundamental care which was not measured. When this lack of measurement of fundamental care is combined with some RNs choosing to withdraw from its delivery, fundamental care could be considered as unseen and unvalued within the hospital culture [21]. The scaffolded hierarchy of HCA tasks focused on physical care provision; the psychosocial and relational interactions known to lead to person-centred care were possible but not guaranteed [10, 21].

For person-centred care to be embedded in ward culture, leaders and the organisation need to provide opportunities and resources, and assess how this philosophy fits with current measurements of safety and quality [21, 40]. In a national inquiry into failures of care that led to avoidable patient deaths, Francis [6] highlighted that the hospital was compliant with regulators at the time of the investigation. The nursing care was, however, found to lack compassion.

There is paradox in the close working co-dependency of the HCA-RN dyad; it has the potential to create a coherent and sustained approach to patient care that ensures their access to expertise and continuity. However, this did not happen organically and could reinforce entrenched task-driven roles. The finding “obs come first” was echoed by Traynor [41] (p6) “act like you have care and compassion but above all keep up with the pace of work”. In this study, there was no indication from those higher in the nursing hierarchy that more patient interaction was required or expected.

### Limitations

Due to the focus on HCAs and their relationship with RNs, there was an absence of the patient voice. Therefore, the impact of dyadic working on the patient experience and their outcomes were not explored. The iterative nature of the observation and the detailed interview data however, marginalised the patient.

The generalisability of the findings to environments outside of the wards used in one hospital in England cannot be assumed. There was however a consistency across the four wards and with previous HCA literature.

### Implications for practice and policy and recommendations for further research

Describing this dyadic relationship provides new insights in to how the HCA and RN negotiate their roles and responsibilities. It has revealed what is identified as supportive of effective and trusting working relationships. The dominance of ward culture and governance observed in this research outweighed the potential for these dyads to support person-centred practice.

These findings reinforce the need to build practice development into the routine work of nursing to address these challenges and competing priorities and encourage collegiate working that is supportive and reflexive [22]. When the HCA-RN dyad worked well, it appeared more likely that the nursing tasks would be carried out on time, with less opportunities for omission or duplication. This implies more efficiency and increased safety for patients if their significance is understood and acted on. There is a possibility that a strong HCA-RN dyad could assist in avoidance of increased deaths on medical and assessment wards [19].

The study overall demonstrates the persistent and enduring challenges of valuing fundamental care as the work of RNs both in education and practice. Further work is needed to test the specific patient benefits of RN involvement in this aspect of care.

## Conclusion

This study found that HCA-RN dyads were integral to effective care delivery and that HCAs work more inter-dependently with RNs than has been previously described. This is important because understanding the construction of how HCAs enact their role and validating the collaborative working between HCAs and RNs provides opportunities to improve the care environment for all. Future work should focus on quantifying the effectiveness of the HCA-RN dyadic relationship on care delivery and inclusion of the patient’s voice.

## Data Availability

Please contact corresponding author.

## Abbreviations

E-obs: Electronic observations
HCA: Health Care Assistant
NEWS: National Early Warning System
NHS: National Health Service
RN: Registered Nurse
UAP: Unlicenced Assistive Personnel
